# Psychometric Features of the Polish Version of TSK Heart in Elderly Patients with Coronary Artery Disease

**DOI:** 10.3390/medicina56090467

**Published:** 2020-09-11

**Authors:** Andrzej Knapik, Józefa Dąbek, Weronika Gallert-Kopyto, Ryszard Plinta, Anna Brzęk

**Affiliations:** 1 Department of Adapted Physical Activity and Sport, Chair of Physiotherapy, School of Health Sciences in Katowice, Medical University of Silesia, 40–055 Katowice, Poland; aknapik@sum.edu.pl (A.K.); rplinta@sum.edu.pl (R.P.); 2 Department of Cardiology, School of Health Sciences in Katowice, Medical University of Silesia in Katowice, 40–055 Katowice, Poland; jdabek@sum.edu.pl; 3 Department of Kinesiology, Chair of Physiotherapy, School of Health Sciences in Katowice, Medical University of Silesia, 40–055 Katowice, Poland; weronika.gallert@gmail.com; 4 Department of Physiotherapy, Chair of Physiotherapy, School of Health Sciences in Katowice, Medical University of Silesia, 40–055 Katowice, Poland

**Keywords:** CAD, TSK Heart, kinesiophobia, physical activity

## Abstract

*Background and objectives*: Recommendations for the control of stable patients with coronary artery disease (CAD) related to an adequate level of physical activity (PA). Practical experience shows that the PA level in most people with CAD is definitely too low in relation to the guidelines. The cause may be psychological factors and among them the fear of movement—kinesiophobia. The aim of this project was to examine the evaluation of psychometric features of the Polish version of the Tampa Scale for Kinesiophobia Heart (TSK Heart), used in people with CAD. *Materials and methods*: The study involved 287 patients with stable CAD: 112 women and 175 men. Age: 63.50 (SD = 11.49) years. Kinesiophobia was assessed using TSK Heart, physical activity (PA)—using the International Physical Activity Questionnaire (IPAQ), and anxiety and depression was examined using the Hospital Anxiety and Depression Scale (HADS). The structure of TSK was examined using principal component analysis (PCA), internal cohesion (Cronbach’s alpha, AC), and content validity was calculated by linear regression. *Results*: PCA showed a three-factor TSK structure. One-dimensionality and satisfactory reliability were found: TSK Heart: AC = 0.878. Kinesiophobia as a predictor of PA: R2 = 0.162 (*p* = 0.000000). Anxiety and depression—TSK: R2 = 0.093 (*p* = 0.00000). *Conclusions*: The Polish version of TSK Heart for cardiac patients is characterized by good psychometric features. The use of it can improve the cooperation of rehabilitation teams for patients with CAD.

## 1. Introduction

Introduction of modern diagnostic methods, development of invasive cardiology, the use of new surgical techniques, more effective medicines, and patient education are aimed at reducing the prevalence of coronary atherosclerosis (coronary artery disease, CAD). However, CAD remains one of the main causes of deteriorating health and increased mortality in Poland, in Europe, and around the world [1,2,3]. In addition to the individual problems of sick people, the occurrence of CAD also entails social costs, especially economic ones—above all, it generates high costs of treatment [4]. The effects achieved in the form of increased survival of patients cause a continuous increase in the need for rehabilitation [5].

Current evidence-based guidelines for the control of stable patients with varying degrees of CAD advise an adequate level of physical activity (PA) [6,7]. It should be provided both by special training programs—cardiac rehabilitation (CR) and daily physical activity [8,9,10,11]. PA integrated with the treatment aims at ensuring the normal physiological and psychosocial functioning of people with CAD as much as possible [12,13]. However, anecdotal evidence and practical experience shows that the PA level in most people with CAD is definitely too low in comparison to the recommendations [9,14,15]. Psychological factors, i.e., acceptance of the disease, disability resulting from it, or changes in lifestyle, have a significant impact on the course and success of cardiovascular disease treatment [16]. According to the researchers, psychological factors are often a key role in achieving intended results in CR, constituting a significant barrier for PA patients with CAD [9,17]. Undertaking activities aimed at increasing physical activity in passive CAD patients will be more effective when PA barriers are diagnosed. Psychologically, they are referred to as “fear of movement”—kinesiophobia.

Originally the term “kinesiophobia” was used to describe anxiety of movement caused by lower back pain [18]. Pain complaints occur in many diseases and are often a barrier to the PA level desired for health. Therefore, a tool for the study of kinesiophobia—Tampa Scale (TSK) began to be applied to other diseases, including to CAD [19,20,21]. Theoretical aspects as well as practical needs prompted Polish language adaptation of TSK Heart. It has been hypothesized that it may have good psychometric properties. Therefore, it was decided to evaluate the psychometric features of the Polish version of TSK Heart, adapted for people with CAD. The dimensionality of the scale, reliability, theoretical, and content validity were analyzed.

## 2. Materials and Methods

### 2.1. Studied Population

The study involved 287 patients, including 112 women (39.02%) and 175 men (60.98%). The research was conducted in the second half of 2017 (from 1 September to 25 November 2017). In this investigation, the sample size was calculated using the sample size calculation for proportions. A margin error of 5% with a confidence level of 95% was used [22]. Study participants were patients at various stages of coronary disease, diagnosed and treated in a Clinical Hospital in the Department of Cardiology in Katowice (Southern Poland). The respondents had to meet the criteria of conscious and voluntary participation in the study and have a sufficient level of mental fitness (contact with the patient, no cognitive disorders) to understand the questions and statements asked in the questionnaire.

### 2.2. Methods

The research was of a cross-sectional study. The aim of the research justified the examination of three aspects: Kinesiophobia, physical activity, as well as anxiety and depression. As a research tool the adopted Polish version of Tampa Scale for Kinesiophobia Heart (TSK Heart), the International Physical Activity Questionnaire (IPAQ) in a short version, and the Hospital Anxiety and Depression Scale (HADS) were used for the purposes of the study. TSK Heart contains 17 statements for which the respondent had four-level Likert scale answers, scored from 1 to 4: “I disagree completely”, “I do not agree partially”, “I partly agree”, “I completely agree.” Statements 4, 8, 11, and 12 had inverted coding. The presented study uses the sum of points assigned to individual statements—the higher the total TSK Heart points, the higher the level of kinesiophobia [23].

#### Language and Substantive Adaptation of TSK Heart

The original version of TSK Heart has been translated into Polish by two translators, in accordance with international guidelines [24]. The next stage included linguistic and substantive adaptation, carried out by a team of experts (translators, cardiologists, physiotherapists, specialists in physical activity). The agreed version was presented to twenty adults of all ages and at various levels of education with a request for full comprehensibility. Three elderly people and those with low educational status required additional explanations, which was an indication that during the research, additional explanation should be provided to people who need it. PA was estimated on the basis of widely used International Physical Activity Questionnaire (IPAQ) in a short version. IPAQ estimates physical activity at three intensity levels: Intense, moderate, and gait during the last week on the basis of patients’ self-report. Frequency (number of days per week) multiplied by time (minutes) and intensity index expressed in MET (Metabolic Equivalent of Work) allowed us to estimate activity in these areas. These indicators are: For intensive activity 8 MET, for moderate 4 MET, for walking 3.5 MET. The sum of intense, moderate, and gait activity allowed us to estimate and express in MET-minutes the total weekly activity of the patient [25]. This indicator was taken into account for statistical analyzes. The following division into groups was adopted due to the level of activity: <600 MET-min/week—passive people, 600–300 METs-min/week—moderately active people, 3000 MET-minutes/week—active people [25]. Patients were asked about the characteristics of their activity related to the week before the hospital stay. To assess mental condition of the patients, the Polish version of the Hospital Anxiety and Depression Scale (HADS) was used [26,27]. This questionnaire evaluates depression and anxiety in patients with somatic disorders. It consists of fourteen statements, which are rated on a scale from 0 to 3 (four-level Likert scale). The sum of points of seven statements creates a subscale regarding anxiety, the remaining seven—depression. Score results are interpreted as follows: 0–7 points—norm; 8–10 points—border values; 11–21 points—disease conditions [26].

The study has been approved by the Bioethical Committee. It is conformed to the Helsinki Declaration. All of the patients provided written informed consent prior to the study, including enrolment and data collection.

### 2.3. Ethical Considerations

The study was conducted according to the provisions of the Helsinki Convention, and also the Bioethics Committee of the Medical University of Silesia Katowice expressed its approval (Decision no.: KNW/0022/KBI/98/15 issued on 7 July 2015). All of the patients provided written informed consent prior to the study, including enrolment and data collection. All of the respondents provided written consent that they agree to take part in this study.

### 2.4. Statistical Analysis

Descriptive statistics were made for the whole group and included sexes. These analyzes included: Mean values, SD, intermediate confidence intervals: ±95% CI. Variable comparisons—based on sexes, were made using one-way ANOVA. Relations between variables were calculated using Pearson’s linear correlation coefficients. The theoretical accuracy and the structure of TSK Heart were examined using principal component analysis (PCA) using varimax rotation, and its internal coherence was examined using the Alfa–Cronbach coefficient (AC). Content validity of TSK Heart was analyzed using linear regression. The assumed statistical significance level was *p* < 0.05. Analyzes were performed using STATISTICA 13.1 package.

## 3. Results

Descriptive statistics (Table 1) indicate a relative homogeneity of the patients regarding age, kinesiophobia (TSK Heart), and level of anxiety. Attention is drawn to the high level of TSK Heart mean values, which are higher than the 37-point criteria adopted by Vlayen et al. [23]. The cut-off point accepted by these authors has been exceeded by 179 respondents, which constituted 62.36% of the total. Including gender: 69 women (61.61%) and 110 men (62.86%). The classification of PA (IPAQ) according to activity levels [26] was as follows: Low level: *n* = 58 (20.21%), mean level: *n* = 164 (57.14%), high level: *n* = 65 (22.65%). Quality assessment of anxiety (HADS)—standard: *n* = 122 (42.51%), border values: *n* = 86 (29.97%), disorders: *n* = 79 (27.53%). Depression (HADS) results were as follows: Norm: *n* = 202 (70.38%), border values: *n* = 61 (21.25%), disorders: *n* = 24 (8.36%). The results are presented in Table 1.

### 3.1. Theoretical Validity of TSK Heart

**Figure 1:**
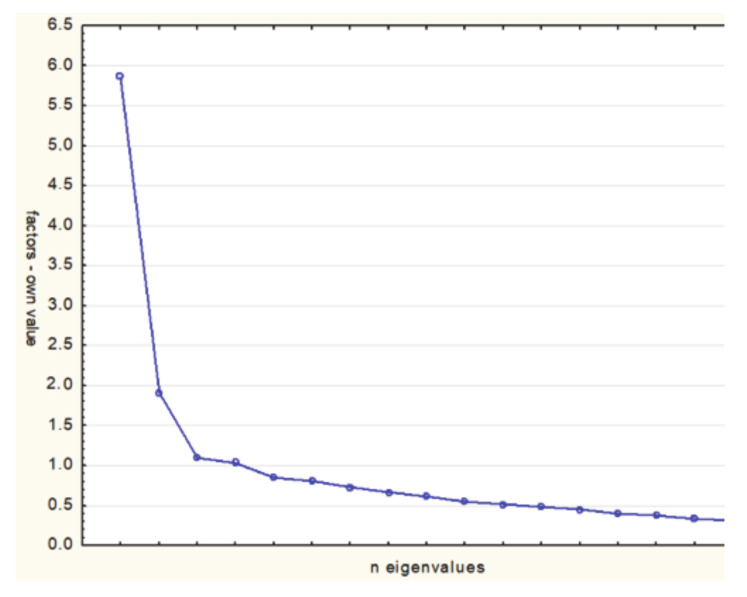
The structure of the Polish version of Tampa Scale for Kinesiophobia Heart (TSK Heart) for patients with coronary artery disease (CAD)—the Catell method.

The initial data analysis using the Bartlett test (*p* < 0.001) and the Kaiser–Meyer–Olkin test (0.756) allowed the use of factor analysis. Factor analysis by the principal component analysis (PCA) allowed to extract, according to the commonly used Kaiser criterion, factors with eigenvalues greater than 1.0. Originally, the assumption was made about the existence of two factors [28]. However, further analysis after varimax rotation showed insufficient correlations of individual items with factors. In addition, the four-factor model did not meet expectations due to insufficient strength of factor loadings. The analysis showed that the three-factor model met the expectations to the greatest extent. The eigenvalue for the first factor was 5.86 (34.35% of the variance), the second factor—1.91 (11.23% of the variance), the third factor—1.10 (6.48% of the variance). In total, these factors explain 52.15% of the variance. The three-factor structure of the TSK Heart scale was confirmed by a diagram made using the Catell method (Figure 1) [29].

After the varimax rotation of factor loadings and removal of <0.3 factor loadings for greater clarity, three domains were specified in this scale. They were defined as: A—disease as a threat, B—physical activity as a threat, C—feeling of being sick. Factor loadings with their assignment to domains are presented in Table 2.

The analysis showed the one-dimensionality of TSK Heart scale. Kline’s criterion determining the level of correlation coefficients: Item—scale (r = 0.4; R^2^ = 0.16) was met in each case. Item–domain correlation coefficients were higher than the required value: r = 0.6 (R^2^ = 0.36). Only the correlation coefficient of the eighth statement was below the required threshold (Table 3) [30].

### 3.2. Reliability

The analyses showed sufficient internal consistency of TSK Heart, fulfilling the Nunnally criterion (AC > 0.7) [31]. The Alfa–Cronbach index was 0.878, and the average correlation between positions: r = 0.301. Half reliability was 0.836 and Guttman index 0.833. (Table 3).

### 3.3. Content Validity

**Figure 2:**
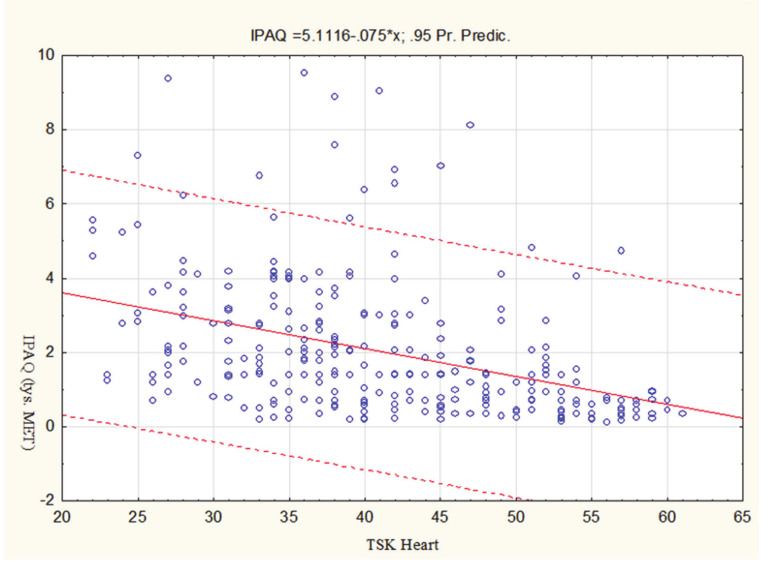
Scatter chart of IPAQ in relation to TSK Heart (±95% CI).

To investigate content validity of TSK Heart, linear regression was applied. First, TSK Heart was adopted as a predictor (independent variable), while the dependent variable was the aggregate IPAQ activity index. The revealed Pearson’s linear correlation coefficient was: r = −0.402. The determination coefficient was: R^2^ = 0.162, (F = 54.98, *p* < 0.0001), and the coefficient B = −0.0750 (Figure 2).

Relationships between anxiety and depression with TSK Heart were also analyzed. In this case, anxiety and depression were independent variables, and TSK Heart—an evolved (dependent) variable. The R2 determination coefficient was 0.093 (F = 14.50, *p* = 0.00000), explaining just over 9% of the variance. Further analysis showed that the B-factor for anxiety was −0.256 (*p* > 0.05); β = −0.095; for depression: B = 0.968 (*p* < 0.0001); β = 0.338 (*p* < 0.0001).

## 4. Discussion

The presented research results seem to confirm the significant impact of TSK Heart on PA in people with CAD. This is indicated by the correlation between TSK Heart and PA, mean TSK Heart results, and the percentage of people with TSK Heart results greater than the Vlayen criteria [23]. The presented data concerning the percentage of people with a high level of kinesiophobia are different from the results obtained by other authors [19,21]. It seems to be a natural consequence of the selection of respondents and lingual and cultural adaptation of the TSK Heart. However, the associations of kinesiophobia with the age of respondents noted by other authors were also confirmed in the presented own research. PA self-assessment results based on IPAQ indicate that the highest number of people presented average activity levels, what is typical for the general population. However, it is worth remembering that the measurement method based on PA self-evaluation was applied. There are studies showing that people tend to notice an excessive assessment of their activity [32]. This would reinforce the thesis that kinesiophobia has a negative impact on health, functional efficiency, and quality of life. TSK Heart has a clearly clinical profile, indicating the disease as the main cause of kinesiophobia [33]. This clinical approach tries to explain the problem of passivity in terms of adaptation to the state of “being a sick person”. The manifestation of adaptation is the application of the strategy of avoiding PA, which is a consequence of perceiving it as a potential threat [34]. The negative influence of erroneous beliefs about the disease on patient’s physical and psychological functioning has been confirmed in research [35]. The presented research results indicate three elements distinguished in the form of presented domains that together have a negative impact on PA. They are psychological categories that can be modified by affecting the cognitive and emotional–cognitive spheres [36]. The complexity of perceiving PA, which may result from both the individual’s own experience, its emotional state, and the general attitude towards activity should be taken into account [37,38]. An important role may also be external factors, such as social and family situation of people with CAD, current level of motor independence, level of social support, and access to organized rehabilitation programs [15,39].

A separate problem is the current psychological disposition of patients, which particularly concerns depression. The presented results indicate its connections with TSK Heart, although due to the limitations of this study, they should be treated in terms of symptoms. It was found that depression is an independent risk factor for the development of coronary heart disease and is associated with a higher risk of death [40,41,42]. Depression also has a significant impact on compliance with medical recommendations, and in the case of patients with severe depression, it is impossible to participate in cardiac rehabilitation programs (CR) [43,44]. It seems that the symptoms of depression may play the role of a moderator in kinesiophobia–PA relations. The article presents widely the need to use the TSK tool in the Polish version, and due to the fact that there are no more reports about this theme, the authors considered this problem to be extremely important in terms of widely defined determinants of successful aging, having been addressed for a considerable time [45,46]. Cultural conditions in the country and the fact that many issues in many regions, especially in the regions of Silesia, are decided by women, which could be important in the results obtained. The limitation of this study is not the size of the cohort. Further research is therefore needed to understand the individual and social impact of the problem—on individual patients and on the quality of healthcare provision. Our research should not be carried out on larger material, but should take into account the possibility of longer observation of CAD patients for further analysis of the test retest. The practical implications of our study can be a valuable tool in the daily practice of evaluating the kinesiophobia of its character of changes in cardiac patients undergoing systematic physiotherapeutic care.

## 5. Conclusions

The Polish version of TSK Heart for cardiac patients is characterized by good psychometric features. The use of it can improve the cooperation of rehabilitation teams in patients with CAD.

## Figures and Tables

**Table 1:** Descriptive statistics of evaluated variables, correlations with TSK Heart, and sex comparison.

Variable	Total			Women			Men			Sex: *p*
	Average (SD)	±95% CI	r-TSK	Average (SD)	±95% CI	r-TSK	Average (SD)	±95% CI	r-TSK	
**Age ^1^**	63.50 (11.49)	62.16 64.83	0.316 ***	63.69 (11.78)	61.48 65.89	0.327***	63.38 (11.34)	61.68 65.07	0.312 ***	0.8338
Duration of the disease ^1^	9.91 (10.28)	8.67 11.16	0.135 *	8.36 (8.69)	6.69 10.03	0.117	10.94 (11.11)	9.22 12.67	0.143	0.0441 *
TSK	41.26 (9.69)	40.14 42.39		41.31 (8.64)	39.70 42.93		41.23 (10.33)	39.69 42.78		0.9470
IPAQ {thous. MET}	2.01 (1.81)	1.80 2.23	−0.402 ***	1.87 (1.73)	1.55 2.19	−0.390 ***	2.11 (1.86)	1.83 2.38	−0.410 ***	0.2771
HADS—anxiety	8.47 (3.60)	8.05 8.88	0.068	9.61 (3.88)	8.88 10.33	0.123	7.74 (3.21)	7.26 8.22	0.037	0.0000 *
HADS—depression	5.84 (3.39)	5.45 6.23	0.293 ***	6.19 (3.88)	5.46 6.91	0.245 **	5.62 (3.02)	5.17 6.07	0.338 ***	0.1647

* *p* < 0.05; ** *p* < 0.01; *** *p* < 0.001; ^1^ years.

**Table 2:** Factor loadings on rotation: TSK Heart structure with domains.

No.	Item	Factor			Domain
		1	2	3	
1	I’m afraid that if I am more active (more moving), it will hurt me.		0.482	0.442	B
2	Always when I was more physically active, I had bigger problems with my heart.			0.652	C
3	I feel bad, I am seriously ill.		0.308	0.649	C
4	Even people with heart disease should be quite physically active.		0.733		B
5	My relatives and friends do not care much about how ill I am.		0.439	0.395	B
6	Because of heart disease, I will not be fully fit until the end of my life.			0.593	C
7	Chest pain is always a sign of serious heart disease.	0.403		0.472	C
8	Symptoms: Oppression, stinging, chest pain are not always dangerous.		0.389		B
9	Due to illness I have to still take care of myself, because I don’t want to hurt myself.	0.545		0.508	A
10	I have to be careful—it is safest to avoid unnecessary movements.	0.427		0.564	C
11	Probably, if I had no trouble with my heart, I would feel much better.	0.410		0.617	C
12	Despite heart disease, I try to be physically active.		0.770		B
13	Every emerging disease symptom indicates that you should immediately stop the current activity—so as not to harm yourself.	0.778			A
14	People with heart disease should not be physically active.	0.357	0.690		C
15	Because of heart disease, I can’t do what others do, because it is too dangerous.	0.590	0.303		A
16	Even when I feel some disturbing symptoms—it does not mean that I have to stop my activity.	0.797			A
17	When you have a heart disease, you should not be physically active.	0.397	0.713		B

Abbreviations: A—disease as a threat; B—activity as a threat; C—feeling of being sick.

**Table 3:** Descriptive statistics of individual TSK Heart items and correlations with scale and domains.

ITEM	Domain	Average (SD)	±95% CI	R^2^-TSK	R^2^-Domain	AC When Deleted
TSK1	B	2.26 (1.05)	2.14–2.38	0.365	0.432	0.759
TSK2	C	2.34 (1.08)	2.22–2.47	0.334	0.434	0.762
TSK3	C	2.37 (0.97)	2.25–2.48	0.373	0.449	0.759
TSK4	B	1.91 (0.93)	1.80–2.01	0.282	0.504	0.764
TSK5	B	2.18 (1.04)	2.06–2.30	0.272	0.372	0.766
TSK6	C	2.64 (1.03)	2.52–2.76	0.449	0.484	0.753
TSK7	C	2.68 (1.00)	2.57–2.80	0.333	0.416	0.760
TSK8	B	2.30 (0.91)	2.20–2.41	0.164	0.295	0.773
TSK9	A	2.71 (0.97)	2.60–2.82	0.423	0.555	0.755
TSK10	C	2.34 (1.06)	2.21–2.46	0.498	0.557	0.762
TSK11	C	3.08 (0.95)	2.97–3.19	0.310	0.416	0.762
TSK12	B	1.95 (0.91)	1.84–2.06	0.271	0.549	0.764
TSK13	A	2.98 (0.95)	2.87–3.09	0.289	0.598	0.764
TSK14	C	1.94 (0.96)	1.83–2.06	0.426	0.372	0.755
TSK15	A	2.71 (0.95)	2.60–2.82	0.423	0.570	0.755
TSK16	A	2.90 (0.98)	2.80–3.00	0.225	0.593	0.767
TSK17	B	1.99 (0.95)	1.88–2.10	0.408	0.497	0.755
